# Designing a Streaming Algorithm for Outlier Detection in Data Mining—An Incremental Approach †

**DOI:** 10.3390/s20051261

**Published:** 2020-02-26

**Authors:** Kangqing Yu, Wei Shi, Nicola Santoro

**Affiliations:** 1 School of Computer Science, Carleton University, Ottawa, ON K1S 5B6, Canada; santoro@scs.carleton.ca; 2 School of Information Technology, Carleton University, Ottawa, ON K1S 5B6, Canada; wei.shi@carleton.ca

**Keywords:** outlier detections, streaming data, data-mining, incremental algorithm, parallel processing, sliding-window

## Abstract

To design an algorithm for detecting outliers over streaming data has become an important task in many common applications, arising in areas such as fraud detections, network analysis, environment monitoring and so forth. Due to the fact that real-time data may arrive in the form of streams rather than batches, properties such as concept drift, temporal context, transiency, and uncertainty need to be considered. In addition, data processing needs to be incremental with limited memory resource, and scalable. These facts create big challenges for existing outlier detection algorithms in terms of their accuracies when they are implemented in an incremental fashion, especially in the streaming environment. To address these problems, we first propose C_KDE_WR, which uses *sliding window* and *kernel function* to process the streaming data online, and reports its results demonstrating high throughput on handling real-time streaming data, implemented in a CUDA framework on Graphics Processing Unit (GPU). We also present another algorithm, C_LOF, based on a very popular and effective outlier detection algorithm called Local Outlier Factor (LOF) which unfortunately works only on batched data. Using a novel incremental approach that compensates the drawback of high complexity in LOF, we show how to implement it in a streaming context and to obtain results in a timely manner. Like C_KDE_WR, C_LOF also employs sliding-window and *statistical-summary* to help making decision based on the data in the current window. It also addresses all those challenges of streaming data as addressed in C_KDE_WR. In addition, we report the comparative evaluation on the accuracy of C_KDE_WR with the state-of-the-art SOD_GPU using Precision, Recall and F-score metrics. Furthermore, a t-test is also performed to demonstrate the significance of the improvement. We further report the testing results of C_LOF on different parameter settings and drew ROC and PR curve with their area under the curve (AUC) and Average Precision (AP) values calculated respectively. Experimental results show that C_LOF can overcome the *masquerading* problem, which often exists in outlier detection on streaming data. We provide complexity analysis and report experiment results on the accuracy of both C_KDE_WR and C_LOF algorithms in order to evaluate their effectiveness as well as their efficiencies.

## 1. Introduction

An *outlier* in a dataset is a data point that is considerably different from the rest of the data as if it is generated by a different mechanism [1]. An interesting property of outliers is that they form minority groups in the dataset, and their patterns can be recognized from their distributions in the datasets themselves rather than relying on a separate training set, which is labelled and expensive to generate in most cases. *Data mining* without labelled data is also called unsupervised learning from a machine learning perspective. A very popular task of unsupervised learning is clustering, where similar data points are aggregated into a cluster repeatedly until all data points are assigned into a group. *Outlier (anomaly) detection* works the other way round. Rather than finding the clusters, which consist of the majority of the data points, it finds spatial data points that do not seem to belong to any clusters.

A very important problem in outlier detection is *masquerading*. Masquerading refers to the fact that outliers may attempt to hide within existing distributions and therefore can hardly be identified [2]. Namely, at different point in time, outliers may exist in different scales and, to properly identify them, the algorithm should be able to process data at different level of magnitude. This would require the updates of hyper-parameters that govern the decisions of outliers to reflect such changes adaptively.

A *data stream* is a continuous, unbounded sequence of data records accompanied and ordered by implicit or explicit timestamps [3]. An important property of data streams is that they are *transient*, which means that data points are only available partially at any given point in time, and random access on the entire dataset is not possible. Moreover, data streams are characterized by *uncertainty* and *concept drift*. *Uncertainty* means that data points are vulnerable to external events (noise) and thus are unreliable [4]. *Concept drift* means that the distribution of data points is not fixed, and it may change over time [5]. Apart from these considerations, when working on applications that process data streams, their temporal contexts need to be considered. In addition, the processing needs to be online or incremental, and data are processed one after the other to leverage the requirement on computational and memory resources. This makes data mining over data streams a challenging task, creating a new research area over the last decades.

With the rapid growth of modern applications, stream programming has become a must in many applications from different fields. Indeed, the increasing popularity of stream programming has led to a new research area compared to a long history of static data processing [6]. This is also true for outlier detection. Outlier detection over data streams can have many applications from different fields, including fraud detections, network intrusion detection, environmental monitoring, and so forth. What is interesting in these applications is that we want to find abnormal behaviours over data streams in *real-time*, with very low latencies. These can be of vital importance in applications such as fraud detections, activity/environment monitoring, networking analysis, and so forth.

Researchers have proposed different solutions to this problem, which aims at detecting outliers in data streams. A popular group of those approaches is called *Distance-Based Outlier Detection in Data Steams (DODDS)* [3,7,8,9], which have been extended from the distance-based outlier detection method first proposed by Knorr and Ng [10] to work in the streaming context. It works by introducing a so-called *sliding-window* in the application and performing learning only on those windowed data. While it performs quite well in some scenarios and also makes real-time results feasible, a big disadvantage of this approach is that the correctness of its results depends largely on the choice of window size and the obsolete data (those expired from the window) are not considered. Other similar techniques exist but most of them fail to address those properties of streaming data, and thus produce results exhibiting poor accuracy.

In this paper, we aim to propose new solutions that overcome aforementioned challenges in streaming context and adopt the *sliding window* technique, but efficiently store in memory a statistical summary of obsolete data, which contributes to the prediction of future data. We first provide a detailed description of our novel algorithm C_KDE_WR, briefly introduced in [11]. This algorithm uses a kernel function to calculate the density for the in-window data and takes advantage of a binned statistical summary to aid with the prediction of incoming data; some of its features include the introduction of a retrospective step and a forgetting factor to overcome the concept drift in data streams. We also provide a complexity analysis on the algorithm design. This algorithm, however, does not solve the masquerading problem; thus, its accuracy could drop drastically should that problem occur. We then present another algorithm that solves this problem. This new approach is based on an existing very popular algorithm called Local Outlier Factor (LOF) [12], which however only works on batched data, and very few works have been introduced to make it work in a streaming context. We show how to modify this algorithm implementing it in an incremental fashion so that it works in a streaming environment, and give theoretical proofs that our solution can process streaming data online in a timely manner without affecting its accuracy. We call this new implementation Cumulative LOF, or C_LOF for short.

For C_KDE_WR, we compare its accuracy with that of the SOD_GPU algorithm presented in Reference [1], which is the state-of-art at the time of writing, using both synthetic and real-life datasets. Both of these algorithms are capable of detecting outliers over streaming data where concept drift may occur. We compare their results using Precision, Recall and F-Score metrics. In addition, we also conducted a t-test with p-value and confidence interval to confirm significance of the improvement. For  C_LOF, in order to demonstrate its ability to overcome masquerading problem, we use synthetic datasets generated from a mixture of Gaussian distributions with same mean but varying variances. We test the accuracy of C_LOF on these datasets and record the ROC and Precision-Recall (PR) curve with various thresholds. In addition, we also calculat the area under the curve (AUC) value for ROC and the Average Precision (AP) value for PR and compare these results with other methods introduced in Reference [13]. Details of these experiments are presented in Section 5.

## 2. Related Works

From the machine learning perspective, most of the outlier detection algorithms can be classified into *supervised*, *semi-supervised* and *unsupervised* categories. Their difference is mainly on the presence of the labelled data. While labelled data can be helpful in building predictive model that imply prior knowledge of data, they also have significant drawbacks when it comes into streaming data.

### 2.1. Supervised Model

*Supervised learning* typically requires building a prediction model for rare events based on manually labelled data (the training set), and use it to classify new events based on this learnt model [14,15]. In other words, the outlier detection problem in this case becomes a classification problem where we are only interested in the minority class whose data deviate largely from the rest. Some machine learning algorithms can be used in the supervised context to detect outliers, such as Support Vector Machines, Neural Network, K-Mean and KNN, and so forth. A recent technique introduced by Harkins et al. [16] takes advantage of replicator neutral network (RNN) to detect outliers. A thing to take note when using supervised method for training is imbalanced data: The predictive models developed using conventional machine learning algorithms could be biased and inaccurate because the number of observations in one class of the dataset is significantly lower than the other. To handle imbalanced data, several methods can be used, including resampling, boosting, bagging [17,18,19,20].

### 2.2. Semi-Supervised Model

To overcome the scarcity of labelled data in supervised learning, *semi-supervised learning* [21,22] only requires a small number of training data with some unlabeled data to obtain better predictions. It is known that applying semi-supervised learning to anomaly detection can improve the detection accuracy [23]. One approach introduced by Jing Gao et al. [24] uses K-mean clustering in unsupervised learning, adding penalties to the objective function for mislabelled data points, and optimizes the overall objective function.

Although efficient in some cases, the main problem of using both supervised and semi-supervised methods is that they work well only with static data, and typically do not fit into the context of dynamic streaming context. In other words, both supervised and semi-supervised methods assume that they have *random access* over the underlying data, while this is not possible for data streams because of its transiency property. Another problem with supervised approaches is that they fail to capture the changes of data pattern since they assume a fixed data distribution and therefore violate the concept drift property of data streams. It is for these reasons that the *unsupervised* algorithms for outlier detection, which we will discuss in the following, have become more popular.

### 2.3. Distance-Based Model

The *distance-based* model introduced by Knorr and Ng [10] was among the very first outlier detection methods that detect outliers on static data. It calculates the pair-wise Euclidian distance between all data and, if one data point has less than *k* neighbours within distance *R*, it is considered an outlier. There are variants of this static distance-based approach. For instance, Ramaswamy et al. [25] proposed a method where an outlier is defined by considering the total number of objects whose distance to its $k^{th}$ nearest neighbour is smaller than itself. Angiulli and Pizzuti [26] introduced a method where an outlier is defined by taking into account the sum of the distances from 1*^st^* up to the $k^{th}$ nearest neighbours. Later on, several methods have been proposed to extend outlier detection onto streaming data [3,7,8,9]. One of the most popular methods uses a *sliding-window* to help with detecting outliers. Based on the benchmark among all DODDS algorithms given by Luan Tran et al. [27], the MCOD algorithm introduced by M.Kontaki et al. [9] appear to have the best performance. In Reference [9], the solution uses a *event-based framework* to avoid unnecessary computations. In addition, to minimize the cost of range query due to the arrival of new objects, it employs evolving micro-clusters to minimize the complexity. The time complexity of this algorithm is guaranteed to be O(nlogk) while maintaining the space complexity to be O(nk), where *n* is the number of data points and *k* refers to the parameter of KNN (K-Nearest Neighbourhood).

Another type of *distance-based* outlier detection model over streaming data is based on the approximation of *probability density function (pdf)*, usually with *Kernel Density Estimator* (KDE) [3]. The distance is measured based on the density of a data point in the estimated pdf around an user defined radius. Sadik et al. [28] first proposed a novel binned implementation of KDE to detect outliers without having to store all observed data and outliers detected if the distance is below a threshold (DBOD-DS). To deal with the *concept drift* of streaming data, they further improved the method by introducing *concept drift* detection module to handle change of distribution in data (A-ODDS) [29]. To handle higher dimension data, the authors proposed a framework, called Orion [30], which addresses all the characteristics of streaming data and looks for projected dimension of high-dimensional data points using evolutionary algorithms. Since DODDS methods only consider a portion of the dataset, the lack of global view on the entire dataset often leads to poor accuracies.

### 2.4. Density-Based Model

The *Density-based* model is another way to detect outlier on static data. The idea is to assign a degree of being outlier (a score) based on the density of local neighbourhood, given some predefined restrictions. A popular example of this approach is Local Outlier Factor (LOF) algorithm [12], on which one of our proposed algorithms is based on. It uses the concept of *reachability* to define the density of data points: the density of each data point is measured by considering the reachability of this data point, in regards to the reachabilities of its neighbours. In Reference [2], D. Pokrajac et al. presented an incremental version of LOF over streaming data. The authors gave theoretical evidence to show that the insertion of new data points as well as deletion of an old data point affects only a limited number of neighbours.

Another popular density-based method is called LOCI (Local Correlation Integral), which uses Multi Granularity Deviation Factor (MDEF) to measure how the neighbourhood count of a particular data point compares with that of the values in its sampling neighbourhood [31].

### 2.5. Probabilistic Model

The *Probabilistic-based* model, also known as *parametric* model, uses the distribution of the data points available for processing. The detection model is formulated to fit the data with reference to the distribution of data [32] and normally models the underlying data using a mixture of distributions (e.g., Gaussian distribution). One of the most popular one used is the *Gaussian mixture model (GMM)* [33,34], where the dataset is fitted into a given number of Gaussian distributions and the model is trained using Expectation-Maximization (EM) algorithm. Each data point is given a formulated score, and data points which have a high score are declared as outliers. These models are usually computational inexpensive but most of them require parameters (i.g., number of clusters) as inputs and they also assume a fixed distribution in dataset, which in most case do not fit into streaming context.

To overcome this problem, in Reference [35], Blei et al. proposed the *Dirichlet Process Mixture Model (DPMM)*, which uses Dirichlet process to infer the number of clusters (components) in dataset. The weight $\pi_{i}$ for each cluster can be described in Dirichlet Process by:

$$\pi_{i}\left(v\right)=v_{i}\prod_{j=1}^{i-1}\left(1-v_{j}\right)\;\;\;q_{\alpha ,\beta }\left(v\right)=\prod_{k=1}^{K-1}Beta\left(\alpha_{k},\beta_{k}\right)$$

where $v_{i}$ follows a Beta distribution, $\alpha_{k}$ and $\beta_{k}$ are variational parameters for each cluster and *K* is the upper bound. The model parameters for base distributions are optimized using Bayesian algorithm and are then tested for convergence by monitoring lower bound on the marginal likelihood. This results in a mixture model where each distribution can be written in exponential-family form to facilitate inference. The scoring is calculated by averaging log likelihood from each distribution using samples generated from their conjugate priors.

In recent years, a least-squared based anomaly detection method was developed by Quinn et al. that also incorporates a hidden Markov model framework in order to identify anomalous subsequences [36]. The method appears to have a faster performance and yet a comparable accuracy compared to other distance-based alternatives.

### 2.6. Auto-Regressive Model

An *autoregressive* or AR model, also known as an infinite impulse response filter or all-pole model, describes the evolution of a variable measured over the same sample period as a linear function of only its past evolution [37]. It is very popular for time series outlier detection and its definition is given by

$$x\left(t\right)=a_{1}\left(t\right)\times x\left(t-1\right)+\ldots +a_{n}\left(t\right)\times x\left(t-n\right)+\xi \left(t\right)$$

where x(t) is the series under investigation, $a_{i}$ are the autoregression coefficients, *n* is the order of the autoregression and ξ(t) is the noise and is almost always assumed to be a Gaussian white noise. Based on this formula, we can estimate the coefficient parameters $a_{i}\left(t\right)$ based on the given time series of x(t),…x(t−n). The model can then be used to predict future time series by defining a threshold, called cut-off limit and the data point is identified as an outlier if it is beyond this threshold.

#### 2.6.1. Deviation-Based Model

The *Deviation-based* model is an approach developed from the *statistical-based* model. In this model, first introduced by Arning et al. [38], an outlier is detected if the feature space of one data point deviates largely from other data points (in local or global set) and the variance is minimized when removing such a point. Aggarwal and YU [39] proposed a technique where a point is an outlier if, in some lower dimensional projection, it is present in a local region of abnormally low density. This method is also efficient with high dimensional data.

#### 2.6.2. Kernel Density Model

The *Kernel density estimator* (KDE) is a non-parametric method to estimate probability density function of random variables [40]. It has become increasing popular in recent year as an efficient way to detect outliers over data streams. The probability density function f(x) is given by:

$$f\left(x\right)=\frac{1}{n}\sum_{n=1}^{n}k_{h_{i}}\left(x_{i}-x\right)$$

where $k_{h_{i}}\left(x\right)$ is the kernel functions with bandwidth $h_{i}$. The kernel functions distribute the occurrence of a data points into its neighbourhood regions and therefore, after observing enough data points, the density function can be curved. Furthermore, the bandwidth can be calculated online using Scott’s rule [40] as new data points are being observed.

Several works have been proposed to use this method for online outlier detection over data streams. A technique inspired from sensor network is mentioned in Reference [41], where it uses a KDE to model the distribution of the sensor data. In Reference [1], Yuni Xia et al. use GPU to accelerate kernel density estimator with helps of *non-overlapping sliding window* and a *statistical binned summary* to detect outliers in high volume and high dimensional data streams. In this method, the outlierness is considered not only based on data points in current window, but also based on historical data that are mined efficiently into bins.

### 2.7. Clustering-Based Model

The *clustering-based* model is another technique to outlier detection over stream data. Two main algorithms exists for clustering-based approaches. One of them is called *K-Mean clustering* [42], which also uses the idea of sliding window and clusters the data in each window. Unlike the distance based approach, the detected outliers are not reported immediately but rather considered as *candidate outliers*. A metric which measures the mean value of each cluster is maintained and carried over to the next window in the stream to further compare with data in other windows. If the candidate outlier passed a given number of windows, it is then identified as *true outlier*. Compared to K-Mean clustering, *K-Median clustering* [43] clusters each chunk of data into a variable number of clusters (from *k* to klog(n) where *n* is the data size and *k* is the KNN parameter), and it passes the weighted medians found in current window into next one for testing outlierness rather than the mean and candidate outliers. Both of approaches require *k* as usersínput, but the number of clusters in K-Median clustering is not fixed.

### 2.8. Other Models

There exist some other approaches that do not fall into any of the previous categories. For example, the *One-Class SVM* method [44], uses Support Vector Machine (SVM) to solve one-class problem. This method uses kernel function to perform dot products between points from input space in high-dimensional space. A hyperplane, also known as decision boundary, is computed by maximizing the margin between the data in the input space and the high-dimensional output space.

Another efficient outlier detection method, especially in high-dimensional data, is *Isolation Forest* [45]. It uses a random forest to recursively ‘isolate’ data points by randomly selecting a feature with a random selected split value. This results in a tree structure and the score of each data point is the path length from the root of the tree to the terminating node. The longer this tree path, it means it is harder to ‘isolate’ this point from the rest. Therefore, points with lower scores are classified as outliers.

## 3. Algorithm C_KDE_WR

In this section, we present in detail the C_KDE_WR algorithm that we have briefly introduced in Reference [11]. C_KDE_WR uses a *sliding window* and *kernel function* to calculate the density for the in-window data and it takes advantage of a binned statistical summary to aid with the prediction of incoming data. More precisely, C_KDE_WR works by calculating approximately the cumulative density function f(x) on the data currently contained in the sliding window as well as the density calculated from data points contained in a statistical binned summary that has been mined from obsolete data. To calculate the density, we use Gaussian kernel estimator as it gives smooth estimation over the entire dataset [1]. To mine the statistical summary, we use a popular technique that bins all obsolete data so that it can be stored efficiently in practice [46]. The density for each bins in statistical summary is also impacted by their forgetting factors, which decay as bins become older (last updated timestamps). If the density for a data point is less than a pre-defined threshold θ, it is considered as a candidate outlier for future inspection. This data point is not defined as true outlier until it has been coined as candidate outlier for a consecutive number of times *R*, which is defined as the rank of the candidate outliers.

### 3.1. Density Estimation

To calculate the density on windowed data, we use a *kernel density estimator* with a Gaussian kernel function f(x) as it gives a smoother estimation [1] and it also works on higher-dimensional data [1]. Since the kernel estimation is a point-based estimation, the model updates dynamically as new data points arrive; therefore, it can solve the *concept drift* problem of data streams. Additionally, as the kernel function f(x) is a probability estimation by its nature, it can also address the *uncertainty* property contained in data streams. The definition of KDE (with Gaussian kernel) is given by Equation (1):

(1) $$k\left(x,x_{i}\right)=\frac{1}{{\left(2\pi \right)}^{D/2}H}exp\left\{-\frac{1}{2}{\left(\frac{x-x_{i}}{H}\right)}^{2}\right\}$$

where k(x) is called the *kernel function*, *D* is the dimension of data points, and

$$H=\left(\begin{array}{cccc} h_{1} & 0 & \cdots & 0\\ 0 & h_{2} & \cdots & 0\\ \vdots & \vdots & \ddots & \vdots \\ 0 & 0 & \cdots & h_{D} \end{array}\right)$$

is a diagonal matrix that denotes the bandwidth of the kernel function. The bandwidth *H* is used to control how much a data point that is far from the current point $x_{i}$ should impact on $x_{i}$. As we use Gaussian kernel, probability of occurrence is distributed to all data points from −∞ to +∞ [40]. We use Scott’s rule [40] to calculate the bandwidth at each dimension based on the following formula:

(2) $$h_{j}=\sigma_{j}n^{1/D+4}$$

where $\sigma_{j}$ is the standard deviation of data points at dimension *j*.

#### 3.1.1. Sliding Window Density Estimation

Due to the unbounded nature of data streams, it is not possible to store all data points in order to calculate the density estimation. In C_KDE_WR, we only store the most recent data points at a regular time interval; expired data points are mined into statistical summary as explained in following section. If we let *W* denote the window size and $T_{0}$ denote the starting time, the window boundaries are therefore $T_{0}+W$, $T_{0}+2W$, …, $T_{0}+jW$ (j>0). To calculate the density in the current window, we substitute all points in current window into the Equation (1), which gives:

(3) $$f_{window}\left(x\right)=\frac{1}{n}\sum_{i=1}^{n}\frac{1}{{\left(2\pi \right)}^{D/2}H}exp\left\{-\frac{1}{2}{\left(\frac{x-x_{i}}{H}\right)}^{2}\right\}$$

where *n* is the number of data points in a sliding window, and *H* is the bandwidth of Gaussian kernel.

However, calculating the density only based on the current sliding window does not give accurate estimate on the overall estimation as no historical data are considered. Therefore, we also need to calculate the density on the statistical summary mined from obsolete data.

#### 3.1.2. Binned Summary Density Estimation

The density of binned summary is calculated slightly differently than of that of the current window (defined in Equation (3)). The bin $B_{i}$ contributes to the density function f(x) by taking into considerations both its mean value vector $M_{i}$ and the number of data points $C_{i}$ in $B_{i}$. If we apply those bins to the Gaussian kernel functions, we derive:

(4) $$f_{bin}\left(x\right)=\frac{1}{C}\sum_{i=1}^{m}\frac{C_{i}}{{\left(2\pi \right)}^{D/2}H}exp\left\{-\frac{1}{2}{\left(\frac{x-M_{i}}{H}\right)}^{2}\right\}$$

where *m* is the number of bins in the binned summary.

When calculating the density of a data point over a binned summary, the freshness of the bin is also considered. We introduce a *forgetting factor* over binned summary that weights each bin according to its freshness. This helps us address the temporal property of the data streams as a more recent bin impacts more than those old ones. To weight each bins, we use *exponential forgetting* as a weight assigning scheme presented in Reference [47], where bin weights are denoted as $\left(\lambda^{n-1},\lambda^{n-2},\lambda^{n-3},\ldots ,1\right)$. If we apply those weights to the density function in Equation (4), we obtain:

(5) $$f_{bin}\left(x\right)=\frac{1}{\sum_{i=1}^{m}\lambda^{m-i}C_{i}}\sum_{i=1}^{m}\frac{\lambda^{m-i}C_{i}}{{\left(2\pi \right)}^{D/2}H}exp\left\{-\frac{1}{2}{\left(\frac{x-M_{i}}{H}\right)}^{2}\right\}$$

To estimate the overall distribution of the probability density function f(x), we define the *cumulative kernel density estimator* function $f_{cumulative}\left(x\right)$ by adding the kernel estimator in the sliding window $f_{window}\left(x\right)$ and the kernel estimator in a binned summary $f_{bin}\left(x\right)$ accordingly. That is,

(6) $$f_{cumulative}\left(x\right)=f_{window}\left(x\right)+f_{bin}\left(x\right)$$

### 3.2. Candidate Outliers and Retrospective

To decide if a data point *x* ia a candidate outlier, we define its *outlier factor* by calculating the inverse of the cumulative density of *x* on the overal kernel density function $f_{cumulative}\left(x\right)$, defined in Equation (6). Thus, the outlier factor $f_{o}$ is defined by Equation (7):

(7) $$f_{o}=\frac{1}{f(x)}$$

We defined threshold $\theta_{threshold}$ on outlier factor $f_{o}$ to cut-off the limit on the precise definition of candidate outlier. The threshold $\theta_{threshold}$ is defined by the average density $p_{avg}$ of all points in current sliding window and the parameter ξ, 0<ξ<1, as follows:

(8) $$\theta_{threshold}=\frac{1}{p_{avg}\xi }$$

Notice that the threshold θ is updated dynamically as new data points arrive in window.

For each detected candidate outlier, we assign a rank *r* which is either incremented or decremented by 1 depending on whether it is a candidate outlier in the current window. If *r* reaches a pre-defined value *R*, it is considered as a true outlier and reported. When *r* reaches zero, it is treated as inlier.

### 3.3. Binned Summary Maintenance

Data points that have been expired from the sliding window are not discarded. Rather than storing all of them, they are mined into a statistical binned summary that can be fitted into limited memory. There are many binned summary mining techniques; we use the one introduced in the literature [46]. There are two steps in bin maintenance. These steps are: (1) Calculate bin index; (2) Update Bin Statistics.

#### 3.3.1. Calculate Bin Index

The bin index is used to indicate which bin a data point belongs to. To calculate the bin index, we assume that the upper and lower bound of all data points in the data stream at each dimension is known a priori. To find the bin index, assume there are *N* data points in the window and each consists of *D* dimensions. For each dimension *j*, we use the upper bound $max(x_{j})$ and lower bound $min(x_{j})$ in order to derive the length of that dimension, and then divide it by a pre-defined value *k* to get its width, Δ:

(9) $$\Delta =[max\left(x_{j}\right)-min\left(x_{j}\right)]/k$$

To find the bin index for each data point $x_{i}$, we first map the input values in each dimension of $x_{ij}$ into interval [0, 1] using the following function:

(10) $$x_{ij}=\frac{x_{ij}-min\left(x_{j}\right)}{max\left(x_{j}\right)-min\left(x_{j}\right)}$$

Then, we encode the data point $x_{i}$ as:

(11) $$<I_{i1},I_{i2},I_{i3},\ldots \ldots ,I_{iD}>$$

where $I_{ij}=x_{ij}/\Delta$. Then, we use the following formula to find the bin index $B_{i}$ for data point $x_{i}$:

(12) $$B_{x_{i}}=\left(I_{iD}-1\right)k^{D-1}+\left(I_{i(D-1)}-1\right)k^{D-2}+\ldots +\left(I_{i2}-1\right)k+I_{i1}$$

where $0\leq i\leq k^{D}$. As we are only interested in the non-empty bins and data in the real-world is generally clustered, the number of actual non-empty bins *m* is generally much smaller than the total number of possible bins $m<<k^{D}$, which does not cause bin number to grow exponentially with the number of dimensions [1].

#### 3.3.2. Update Bin Statistics

For each bin, we maintain its bin count (noted as $C_{i}$) that denotes the number of data points that have fallen into this bin and its aggregate mean value vector (noted as $M_{i}=<\mu_{i1},\mu_{i2},\ldots ,\mu_{iD}>$), which comprises of the average mean value $\mu_{ij}$ at each dimension *j*. Additionally, we also maintain the mean value vector μ and the standard deviation Σ over the entire dataset until now.

To update the bin statistic when processing the $n^{th}$ window, once the previous ${\left(n-1\right)}^{th}$ windows of obsolete data have been processed and aggregated into binned summary, we first group all data points in the current window by its bin index *i* calculated using Equation (12); we then derive the mean value vector $\mu_{i}^{n}$ and bin count $c_{i}^{n}$ for each bin $B_{i}$ at index *i*. We then update the cumulative mean value vector $M_{i}^{n}$ and bin count $C_{i}^{n}$ at $n^{th}$ window using:

(13) $$M_{i}^{n}=\frac{c_{i}^{n}\mu_{i}^{n}+C_{i}^{n-1}M_{i}^{n-1}}{c_{i}^{n}+C_{i}^{n-1}}$$

(14) $$C_{i}^{n}=c_{i}^{n}+C_{i}^{n-1}$$

where $C_{i}^{n-1}$ denotes the total number of data points that fall into bin $B_{i}$ up to the ${\left(n-1\right)}^{th}$ window and $M_{i}^{n-1}$ denotes the mean value vector of data points in bin $B_{i}$ up to ${\left(n-1\right)}^{th}$ window. Once we updated bin $B_{i}$, we also need to update its *last-updated timestamp* by setting it to the timestamp of the most recent data point in that bin from the $n^{th}$ window. This is done in order to derive its *forgetting factor* as shown in Equation (5).

### 3.4. Complexity Analysis

As algorithm C_KDE_WR is composed of two main parts, *density estimation* and *bins maintenance*, we analyze their time complexities separately.

The density is estimated for each *query point* over *reference points*. The query points are those points from the current sliding window plus the candidate outliers from previous windows; the reference points are those in the sliding window plus bins in binned summary. If we denote the number of data points in current window as *N*, the dimension of data points as *D*, the number of candidate outliers from previous window as *C*, the number of bins currently in system as *M*, and the time complexity to apply the Gaussian kernel function defined in Equation (1) as $T_{kernel}$, then the time required for density estimation is given by:

$$T_{density\_estimation}=\left(N+C\right)\left(N+M\right)T_{kernel}$$

since the density estimation is linear over all query points and, for each query point, it is linear over all its reference points. Notice that the time complexity for running Gaussian kernel function $T_{kernel}$ is also linear over the dimension *D* of data points; we can therefore expand the $T_{kernel}$ as:

$$T_{kernel}=D\;\cdot \;T_{kernel\_d},$$

where $T_{kernel\_d}$ is the time complexity to apply the Gaussian kernel function defined in Equation (1) at each single dimension. Therefore:

**Theorem** **1.** *The complexity for density estimation in algorithm C_KDE_WR is bounded by:*

$$T_{density\_estimation}=\left(N+C\right)\left(N+M\right)D\;\cdot \;T_{kernel\_d}=O\left(D\;\cdot \;N^{2}\right)$$

*where C and M are independent of both N and D and they are treated as constants.*

The bins maintenance consists of two steps—*calculate bin statistics* and *update binned summary*. We first need to calculate the bin index for each point in window, which gives a linear time complexity over data dimension *D* for each single point and thus a linear time complexity over the total size *N* of the data points. The overall complexity is therefore bounded by O(D·N). We then group data points by their bin index, which requires time linear in *N* as each record would need to be traversed; finally we perform aggregations on each of these bins to derive $\mu_{i}^{n}$ and $c_{i}^{n}$, which also uses linear time over D·N as each dimension of a single data record need to be scanned for all data points regardless of which bins they belong to.

Once we get the all statistics, we update the global bin, as mentioned in Equations (13) and (14), which takes only constant time for each mined bin. In the worst case scenario, each data point from current window is scattered into different bins, in which case, after mining over all data points in the window of size *N*, we get *N* mined bins to update. That is, this step requires linear time of data size *N* in the worst case. Therefore:

**Theorem** **2.** *The complexity for bin maintenance in algorithm C_KDE_WR is bounded by:*

$$T_{bin\_maintenance}=T_{in-window}+T_{out-of-window}=aD\;\cdot \;N+bN=O\left(D\;\cdot \;N\right),$$

*where a,b are constants.*

## 4. Algorithm C_LOF

In this section, we introduce our second algorithm, Cumulative Local Outlier Factor (C_LOF), that is based on a very popular outlier detection technique, called Local Outlier Factor (LOF) [12], which however only works on batched data. In C_LOF, we use a sliding window to maintain *active data points* and incrementally update their proximities as new data arrive or old data expire; this process works exactly in the same way as discussed in the literature [2]. Furthermore, we also keep statistical summary of historical data to help predict the proximities of active data points, which gives novelty to this algorithm. Expired inlier points are clustered as *virtual data points* and combined with active points in the current window to execute algorithm LOF incrementally. This is done in order to address the concept drift issue in data streams. Algorithm C_LOF can also overcome the masquerading problem in outlier detection. Moreover, to incorporate the temporal context in data streams, we introduce the forgetting factor λ on all virtual points as we have done in the binned summary of C_KDE_WR Equation (5). In the following, we provide the details of C_LOF procedures.

### 4.1. Local Outlier Factor

Let us first look at the classical LOF algorithm proposed by Breunig et.al in 2000 [12]. The main idea of algorithm LOF is to assign to each data point a degree (or score) of being outlier; this degree is called *Local Outlier Factor (LOF)* of the data point. The metric measures the density of a data point compared to its neighbourhood (K-nearest neighbours). The computing of *LOF*s for all data points typically comprise of the following steps [12]:

1. For each data point *p*, compute its *k*-distance(p), i.e. the distance to its $k^{th}$ nearest neighbour.
2. For each data point *p*, find its *k*-distance-neighbourhood of *p*, which contains every object *q* whose distance to *p*, noted as d(p,q) is not greater than *k*-distance(p).
3. For each data point *q* in the *k*-distance-neighbourhood of *p*, calculate its reachability distance with respect to data record *p* as follows:
  (15) reach−dist(p,q)=max(d(p,q),k−distance(q))
4. For each data point *p*, calculate its *local reachability density (lrd)* of *q* as inverse of the average reachability distance over *k-nearest neighbour* of *p*:
  (16) $$lrd\left(p\right)=\frac{1}{\sum_{k\in knn(p)}reach-dist\left(p,q\right)/k}$$
5. Finally, for each data point *p*, calculate its LOF as ratio of average lrd over *k-nearest neighbour* of *p* and lrd of *p* itself
  (17) $$LOF\left(p\right)=\frac{\frac{1}{k}\sum_{k\in knn(p)}lrd\left(p\right)}{lrd(p)}.$$

We assume that the distances between each pair of data points are different and, in the original publication, *k* was also named *MinPts*, which means the minimum number of data points in a cluster in order to consider this cluster as inliers [12]. The outlierness is detected once the *LOF* value of a data point *p* deviates largely from the average value of *LOF* in the population. This is often controlled by the hyper-parameters that defined the maximum threshold θ that the algorithm can tolerate (as inliers).

### 4.2. Incremental LOF

To address the challenge of applying LOF over data streams, an incremental LOF algorithm was proposed in Reference [2]. The incremental LOF works by constantly maintaining *k-distances*, *lrd* and *LOF* values for all existing points and incrementally updating these values whenever a new data point is inserted or an obsolete data point is deleted. Since the static LOF algorithm has time complexity of O(N·logN), if we apply LOF algorithm *iteratively* after observation of *N* data points, the algorithm gives a $O(N^{2}\;\cdot \;logN)$ time complexity [12]. In Reference [2], they proved theoretically that the insertion and deletion of data points actually only affect a limited number of existing data points (neighbours) rather than the total number of data points in dataset, and therefore the total complexity of incremental LOF algorithm is bounded in practice by O(N·logN). In addition, they also illustrated that the result of applying their incremental LOF algorithm is the same as the result of applying the static version of LOF algorithm after receiving *N* data points, and it is also independent of the order of the insertions.

### 4.3. Update Operation

We design our algorithm C_LOF based on the incremental LOF algorithm; in particular the insertion and deletion operations are as in the original paper in Reference [2]. However, in C_LOF, we introduce an update operation which is performed when positions of some points within the dataset have changed. This operation can become complicated as the change of position of a particular point within the dataset may cause *k-distances* of some points in the dataset to decrease while it can also cause *k-distances* of other points to increase. Indeed, the change of position of any point may break the *K-NN* relationship that has been previously established among data points. Particularly, the $k^{t}h$ neighbour of an updated point $p_{n}$ may change due to the change of position of other points in the dataset. The safest option for updating a point $p_{n}$ is to first perform the *delete operation* on point $p_{n}$, followed by an *insert operation* on point $p_{n}$ based on its new position. While this guarantees the correctness of the update operation, the execution maybe very time consuming, since large amount of K-NN and K-RNN range queries need to be executed for insert and update operations. In addition, when the change of position of a point $p_{n}$ is very tiny, it is unlikely to cause the *K-NN* relationships among data points to change and therefore executing delete and insert operations result in many redundant range queries. Therefore, we need to simplify the update operation when the change of position is tiny.

For the update operation, we assume that, when the change of position (in terms of Euclidian distance) of a point $p_{n}$ is within a threshold ε, for all point $p^{\prime }$ in the dataset, the $k^{th}$ nearest neighbour of point $p^{\prime }$ would not change. Therefore, the *k-distances* only changes for those points $p_{c}$ whose $k^{th}$ nearest neighbour is point $p_{n}$, noted as $r\text{-}k^{th}\left(p_{n}\right)$, and point $p_{n}$ itself. For points $p_{c}\in r-k^{th}\left(p_{n}\right)$, their *k-distances* should be updated based on the new Euclidian distances between $p_{c}$ and $p_{n}$. We also need to consider the *reachability-distances* that have been affected by such changes of *k-distances* and re-calculate lrd values for those points affected just as in insertion and deletion operations. For point $p_{n}$ itself, we need to re-calculate its *k-distance* to its $k^{th}$ neighbour since its position has changed. Since $p_{n}$’s *k-distance* is updated, the *reachability distances* between all points in its *k* neighbours, $k\text{-}NN(p_{n})$ to point $p_{n}$ have also been updated; therefore, for all points $q\in k\text{-}NN(p_{n})$, if *q* satisfies $p_{n}\in k\text{-}NN\left(q\right)$ or $q\in k\text{-}RNN(p_{n})$, then its lrd values should be updated. As a result of change of position of $p_{n}$, the Euclidian distances from every points to $p_{n}$ have changed. Therefore, the reachability distances from any point *q* such that $q\notin k\text{-}NN(p_{n})$ to $p_{n}$ changes; thus, the lrd value changes for those points of *q* such that $q\in k\text{-}RNN(p_{n})$ and $q\notin k\text{-}NN(p_{n})$. Apart from them, the lrd value of point $p_{n}$ itself need to be updated since the Euclidian distances to every of its *k* neighbours have changed. For update of *LOF* values, it is the same as insertion and deletion operations. Algorithm 1 depicts the update operation in details.
**Algorithm 1 Incremental LOF Update** (Dataset S, Point $p_{n}$)**if**$\Delta p_{n}<\varepsilon$**then**    $S_{update\_k\_distance}$ = **Compute** $r\text{-}k^{th}\left(p_{n}\right)\cup p_{n}$;    Update(S, $p_{n}$);    $S_{update\_lrd}$ = **Compute** $\left\{\left[k\text{-}RNN\left(S,p_{n}\right)-k\text{-}NN\left(S,p_{n}\right)\right]\right\}\cup p_{n}$;      **for all** $p\in S_{update\_k\_distances}$ **do**        **Compute** k-distance(S,p);          **for all** q∈k-NN(S,p) **do**           **if** p∈k-NN(S,q) **then**                 reach-dist(q,p)=k-distance(S,p);                 $S_{update\_lrd}\cup q$;           **end if**        **end for**    **end for**  $S_{update\_LOF}=S_{update\_lrd}$;    **for all** $p\in S_{update\_lrd}$ **do**        **Compute** k-NN(S,p);          **for all** q∈k-NN(S,p) **do**                **Get/Compute** reach-dist(p,q) using Equation (15);          **end for**        **Update** lrd(p) using Equation (16);        $S_{update\_LOF}\cup k\text{-}RNN\left(p\right)$;    **end for**    **for all** $p\in S_{update\_LOF}$ **do**      **Get** lrd(p);        **for all** q∈k-NN(S,p) **do**                **Get** lrd(q);        **end for**      **Update** LOF(p) using Equation (17);    **end for****else**    **Deletion**(S, $p_{n}$);    **Insertion**(S, $p_{n}$);**end if**

### 4.4. Maintenance of Active Data Points

The first step of our algorithm C_LOF is the maintenance of active data points in the current sliding window. The maintenance of active points starts when a new data point is fed into the C_LOF algorithm. Every new data point coming after the first window runs the algorithm incrementally as shown in Section 4.2. More precisely, when a new data point arrives, we need to first delete the oldest data point in the window by performing the deletion operation and then insert the new one by performing the insertion operation. Therefore, we need to maintain a queue in the sliding window so that data points can arrive and depart in a FIFO manner. Algorithm 2 describes how our active data points in the sliding window are maintained upon arrival of each new data point.
**Algorithm 2 Sliding Window Maintenance** (Queue window, Point $p^{new}$)**if**|window|<W−2**then**    $window.enqueue(p^{new}$);**else**    **if** |window|==W−2 **then**        $window.enqueue(p^{new}$);        **Non Incremental LOF**(window.active_points);    **else**        **if** |window|==W−1 **then**        $window.enqueue(p^{new}$);        **Incremental LOF Insertion**(window.active_points, $p^{new}$);        **else**        $p^{old}=window.dequeue\left(\right)$;        **Incremental LOF deletion**(window.active_points, $p^{old}$);        $window.enqueue(p^{new}$);        **Incremental LOF Insertion**(window.active_points, $p^{new}$);        **end if**    **end if**    Computer threshold θ;**end if**

### 4.5. Maintenance of Virtual Data Points

Step two of algorithm C_LOF is the maintenance of virtual data points. Instead of throwing every obsolete data away right after they expire, we cluster them incrementally and store each cluster as a virtual data point with its total number of data points it contains. The virtual data points contain the position information and the proximity information (e.g., k-distances, lrd and *LOF* values) about all data points that fall into this cluster previously and they can affect the proximity information of active data points. More importantly, at each iteration, the calculation of virtual data points depends only on their previous states and the total number of points in the cluster. It does not depend on the whole data points in that cluster; therefore, they can be computed incrementally.

The maintenance of virtual data points starts whenever a data point expires from the active window and that point is NOT an outlier. It first needs to execute the clustering algorithm that runs incrementally to mark all active points that belongs to the same cluster as this one, and then merge its position and proximity information into the virtual data point of the cluster it belongs to. The cluster that this expired data point belongs to should (ideally) be marked by previous points that expired. Once we decide to which cluster each expired data point belongs, we first update the position of that cluster incrementally by calculating its average value, based on its previous position, the number of data points in that cluster and the current position of this expired point (as shown in Equation (18)).

(18) $$p_{virtual}^{new}=\left(p_{virtual}^{old}*count^{old}+p^{expired}\right)/\left(count^{old}+1\right).$$

If the expired data point has not been marked by any previous expired points, it forms a new cluster on its own, and the point just stays as is, except that its status has changed from active to virtual. That virtual point becomes the first point of that cluster along with its proximity information copied directly from its previous values as active point.

The same goes for updating the proximity values for virtual data point. They are calculated based on the average *k-distance* and *lrd* values above all points in that cluster rather than by calculating their neighbourhoods. Therefore, for each cluster (virtual point), we only need to keep the average positions, average *k-distance* values, average *lrd* values and the *count* of data points in that cluster in memory. Once the information of a virtual data point is updated, we need to calculate the k−distances, lrd and *LOF* values of active data points that are affected by such changes and update them accordingly. This can be achieved by performing the update operation described in Section 4.3. Before discarding this expired data point, we also need to work out the weighted lrd values for each virtual point by applying forgetting factor in the same fashion as in C_KDE_WR, shown in Equation (19) and these are the actual lrd values of virtual data points that are used for computation. After all these steps, we can safely discard this point as its information has already been incorporated in that cluster.

(19) $$lrd_{projected}^{v}=\lambda^{n-i}\;\cdot \;lrd^{v}.$$

### 4.6. Complexity Analysis

We first discuss what happens when each individual point $p^{new}$ is fed to the algorithm. The situation would depend on the number of data points, noted as *N* that are currently in the system. If N<W−2, where *W* is the number of data points contained in each window, the time complexity of this operation would only consist of the time to insert new data point to data stores of each components, noted as $T_{insert}$. If we use a KD-Tree data structure, the average time for $T_{insert}$ would be O(logN). Insertions to other components would take constant time if we store them using hashtables. Therefore, the time complexity of C_LOF when no LOF calculation actually happens would be simply:

$$T_{C\_LOF}^{N<W-2}=T_{insert}=O\left(logN\right).$$

When N=W−2, C_LOF inserts the point $p^{new}$ as before and trigger the static LOF algorithm that runs in batch mode. Since the time complexity $T_{LOF}$ of the static LOF algorithm is O(N·logN) is [12], the time complexity to process initial *N* data points is given by:

$$T_{C\_LOF}^{N=W-2}=T_{insert}+T_{LOF}\leq a\;\cdot \;logN+b\;\cdot \;N\;\cdot \;logN=O\left(N\;\cdot \;logN\right)$$

where a,b are constants.

When N=W−1, C_LOF starts processing each future points incrementally. The time complexity of C_LOF algorithm in this case would be exactly the same as the insertion operation in incremental LOF algorithm, noted as $T_{LOF\_insert}$. The insertion operation in incremental LOF has time complexity $O(k\;\cdot \;F\;\cdot \;T_{kNN}+k\;\cdot \;F\;\cdot \;T_{kRNN}+F^{2}\;\cdot \;k+T_{insert})$ [2], where *k* is the predefined number of nearest neighbours of point $p^{new}$, *F* is the maximum number of reverse nearest neighbours of $p^{new}$, which is proportional to *k*, and $T_{kNN}$ and $T_{kRNN}$ are the time complexities of k−NN and k−RNN range queries respectively. For KD Tree, time complexities of these range queries can be reduced to O(logN), as compared to the naive brute-force approach which would require $O(N^{2})$ time complexity. If we denote F=ck, the time complexity of C_LOF algorithm when N=W−1 is bounded by:

$$T_{C\_LOF}^{N=W-1}=ck^{2}\;\cdot \;\left(T_{kNN}+T_{kRNN}\right)+c^{2}k^{3}+T_{insert}\leq 2ack^{2}\;\cdot \;logN+b\;\cdot \;logN+c^{2}k^{3}=O\left(k^{2}\;\cdot \;logN+k^{3}\right)$$

where a,b,c are constants.

When N=W, C_LOF first removes the oldest data point $p^{old}$ from window by performing the delete operation. Depending on the status of $p^{old}$, C_LOF triggers the cluster maintenance steps. The deletion operation when N=W has the same time complexity as the insertion operation when N=W−1. If no cluster maintenance steps are triggered (if $p^{old}$ is an outlier), C_LOF just runs the insertion operation after performing the deletion operation. Therefore the time complexity in this case would be:

$$T_{C\_LOF}^{{\left(N=W\right)}^{best}}=T_{LOF\_insert}+T_{LOF\_delete}=2T_{LOF\_insert}=O\left(k^{2}\;\cdot \;logN+k^{3}\right).$$

If the cluster maintenance steps are triggered ($p^{old}$ is an inlier), beside the insertion and deletion steps, C_LOF first need to run incremental clustering. The time complexity of incremental clustering, noted as $T_{clustering}$, is dependant on the clustering algorithm. The DBSCAN algorithm has an average runtime complexity of O(N·logN) and worst case runtime complexity of $O(N^{2})$ [48]. Using that procedure in our algorithm, $T_{clustering}$ will have the same bound in the worst case.

$$T_{clustering}=O\left(N^{2}\right)$$

The update of virtual point and its proximity information takes only constant time; the time used by the update operation, noted as $T_{LOF\_update}$, it is at most twice that required by the insertion operation.

$$T_{LOF\_update}\leq 2T_{LOF\_insert}=O\left(k^{2}\;\cdot \;logN+k^{3}\right).$$

At the end, C_LOF applies the forgetting factor, calculates the projected lrd values for all virtual points, and updates the *LOF* values of the affected active points. Let *m* denote the number of virtual points currently in the system; by definition of *LOF*, $m<\frac{N}{k}$ in worst case. For each of these virtual point, we need to run the k-RNN range queries and find its reverse neighbours. If we denote the number of these reverse neighbours as F=ck where *c* is a constant, then for each of these reverse neighbours, their *LOF* values need to be updated based on Equation (17), which has exactly the same time complexity as k-NN range queries. Assuming k-NN and k-RNN both have the time bound of O(logN) and $m=\frac{N}{k}$, the total time complexity to apply the projected lrd values on all virtual points, noted as $T_{projected\_lrd\_update}$ would be:

$$T_{projected\_lrd\_update}\leq m\;\cdot \;\left(logN+ck\;\cdot \;logN\right)=O\left(N\;\cdot \;logN\right).$$

**Theorem** **3.** *The time complexity of C_LOF after observing each individual data point in case N=W and where cluster maintenance steps are necessary has an upper bound of:*

$$T_{C\_LOF}^{{\left(N=W\right)}^{worst}}=T_{LOF\_insert}+T_{LOF\_delete}+T_{clustering}+T_{LOF\_update}+T_{projected\_lrd\_update}=O\left(N^{2}+k^{2}\;\cdot \;logN+k^{3}\right).$$

This bound is for the worst case. As $T_{clustering}$ is O(NlogN) on average, the average time complexity of $T_{C\_LOF}$ can be reduced to $O(NlogN+k^{2}\;\cdot \;logN+k^{3})$ in most cases.

Consider now the case when *n* data points are fed into the algorithm and each of them is processed individually. We first discuss the initial *n* data points that are first fed to our algorithm, where n=W−1. In that case, the first n−1 points do not trigger any update and they only need to be stored in the sliding window. The $n^{th}$ data point triggers the static LOF algorithm that runs in batch mode. Since the LOF algorithm has time complexity of O(n·logn) [12], the time complexity to process the initial *n* data points is therefore given by:

$$T_{C\_LOF}\left(n\right)=\sum_{i=1}^{n-1}T_{insert}+T_{LOF}=O\left(n\;\cdot \;logn\right).$$

where n=W−1. Later on, whenever a new point is inserted, it triggers the C_LOF algorithm to run incrementally. As discussed previously, each insertion of individual data point has an upper bound of $O(N^{2}+k^{2}\;\cdot \;logN+k^{3})$, where *N* here would be equal to the number of data points *W* in each window. Therefore, in a count-based window, where *W* is a constant, we have:

**Theorem** **4.** *The time complexity of C_LOF after observing n data points is:*

$$T_{C\_LOF}\left(n\right)=\sum_{i=i}^{n}T_{C\_LOF}\leq nW^{2}+nk^{2}\;\cdot \;logW+nk^{3}=O\left(nk^{3}\right)$$

## 5. Experiments and Results

We present the experimental results on both proposed algorithms. Experiments have been performed over both synthetic and real-life datasets. For C_KDE_WR, we compare its accuracy with the method proposed in the literature [1] using both synthetic and real-world datasets. To measure the accuracy, we use different metrics including Precision, Recall and F-score, and so forth. We also performed a t-test to further confirm that the accuracy of C_KDE_WR out-performed SOD_GPU due to our novelties introduced. For C_LOF, we measure its accuracy on different parameter settings in synthetic datasets. In addition, we draw the Receiver Operating Characteristics (ROC) curve (true positive rate against false positive rate) and Precision-Recall (PR) curve on C_LOF at various threshold settings. We want to demonstrate that C_LOF is efficient in the streaming context even with *masquerading* problem.

### 5.1. Datasets

#### 5.1.1. Synthetic Datasets

For C_LOF algorithm, we only measure its accuracy on synthetic datasets using Gaussian mixture model with outlier points generated using uniform distribution given a range. We chose the Gaussian mixture model because it does not conform to a fixed distribution and its data pattern can change over time to simulate real data streams. We conducted the experiments in two different settings. In one setting, we want to measure the ability to detect concept drift of our model and we therefore generated two 2-dimensional Gaussian distributions with different means but same variances (noted as Synthetic 1 dataset in experiments). In the second setting, we generate two 2-dimensional Gaussian distributions with same mean but different variances (noted as Synthetic 2 dataset in experiments). Each of the Gaussian distribution consists of 200 data records and they may appear at different point in time. 20 outlier points are generated randomly within a range specified that deviate largely from these distributions. Data records are fed to the algorithm 100 at a time so the window size is set at 100.

For C_KDE_WR algorithm, we also generate synthetic data from Gaussian mixture model using similar settings as in C_LOF. We generate 10,000 data samples from eight different dimension settings (from 2 to 9) using Gaussian mixture with different means but same variances. These points were considered as inliers and are ordered by the distributions that they belong to. 100 outlier points were generated uniformly and shuffled into inlier points in a random order in each dimension setting.

#### 5.1.2. Real-World Datasets

Real-world datasets are only measured on C_KDE_WR algorithm. We use two real-world datasets obtained from UCI machine learning library (http://archive.ics.uci.edu/ml/datasets.html): KDDCup99 network dataset for the intrusion detector learning task, and Covertype forest cover dataset for cover type prediction task in forest, which are both designed for classification tasks. In order to make them suitable for outlier detection task, we chose classes with minority instances as outlier points (i.e., less than 10% occurrence). Specifically, for KDDCup99 dataset, points belong to *normal*, *smurf* and *nepturn* classes as considered as inliers. All other classes are considered outliers. For Covertype dataset, points belonging to class *Spruce-Fir* and *Lodgepole Pine* are considered inliers. Other classes are considered as outliers. We take some preprocessings on both real-world datasets and randomly chose 10,000 samples based on the proportion of each class, where outlier points are uniformly distributed.

### 5.2. Test Environment

We implemented the C_KDE_WR algorithm using NVIDIA CUDA framework (Compute Unified Device Architecture: https://developer.nvidia.com/cuda-zone) to parallelize the computations for kernel density estimations and takes advantages of Apache Flink framework (https://flink.apache.org/) to simulate the streaming environment for C_LOF. All experiments were performed on a server with Ubuntu 16.04 operating system, equipped with an Intel 3.3 GHz quad-core CPU and 64 GB host memory, along with an NVIDIA GTX 1080 Ti GPU (6.1 compute capability). The CUDA runtime version used was 9.2 and the Flink version was 1.7.2. We used Numba JIT compiler (http://numba.pydata.org/numba-doc/latest/index.html) to implement C_KDE_WR algorithm in CUDA. Numpy (https://numpy.org/) library was used to implement our C_LOF algorithm in an incremental fashion.

### 5.3. Evaluation Criteria

To evaluate the accuracy, we use the same metrics as the binary classification task in machine learning. Outlier detection can be thought of as a special type of binary classification task since each data point needs to be classified as either inlier or outlier. The only difference is that the dataset used for outlier detection is hugely unbalanced. In order to measure accuracy of C_KDE_WR, we use Precision, Recall and F-Score, which is widely used for accuracy evaluation in binary classification.

Precision is defined as the number of correctly detected outliers (true positives) divided by the total number of detected outliers (true positives + false positives). Recall is defined as the number of correctly detected outliers divided by the total number of outliers in the dataset (true positives + false negatives), and F-Score is defined as:

$$F_{score}=\frac{2\times precision\times recall}{precision+recall}.$$

We compare accuracy of C_KDE_WR with the SOD_GPU algorithm proposed in Reference [1] on both synthetic and two real-life datasets as mentioned previously. We also performed a t-test between these two algorithms and recorded the p-value, confidence interval and variance to further support that our C_KDE_WR algorithm has improved over its counterpart in terms of accuracy.

For C_LOF, we measure its accuracy against different parameters of *k* on two synthetic dataset settings generated from mixture of Gaussians as mentioned before: One is fixing on variance but varying mean, the other is fixing on mean but varying on variance. We want to show that C_LOF can detect outliers in data streams with both concept drift and masquerading problems as mentioned earlier. To further prove its efficiency, we also draw the Receiver Operating Characteristics (ROC) curve and Precision-Recall (PR) curve of C_LOF at varying threshold settings, using the LOF score of each trained data point. The positive class denotes outliers and negative class represents inliers. Finally, we calculate Area Under the Curve (AUC) for ROC using the trapezoidal rule and summarize PR curve using Average Precision (AP) as the weighted mean of precisions at each threshold, given by:

$$AP=\sum_{n}\left(R_{n}-R_{n-1}P_{n}\right),$$

where $P_{n}$ and $R_{n}$ are precision and recall at the $n_{th}$ threshold.

### 5.4. Accuracy Evaluation for C_KDE_WR

**Figure 1:**
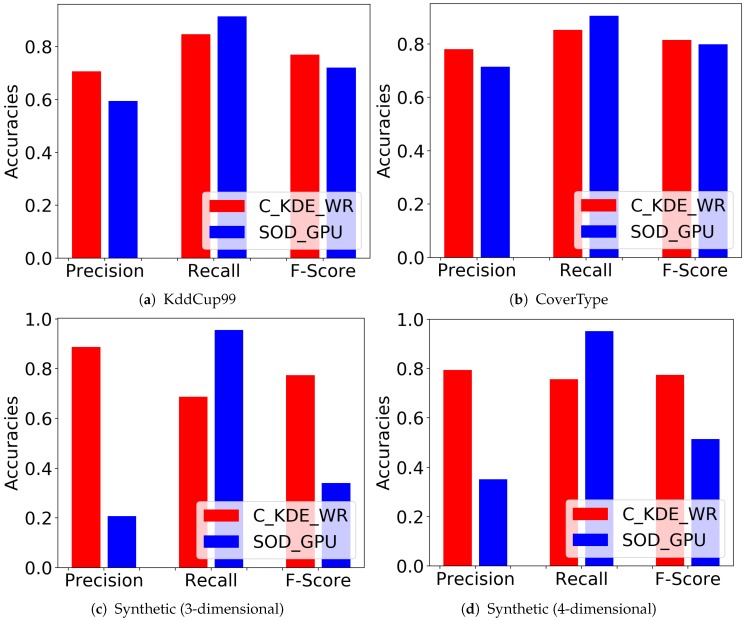
Average accuracy of C_KDE_WR on KddCup99 dataset (**a**), CoverType dataset (**b**), and Synthetic datasets (**c**,**d**).

We set ξ to 0.1 and *k* to 100 in C_KDE_WR. Three retrospects (R=3) are required to finalize a true outlier detection. 0.5 is selected as forgetting factor λ and the window size is set at 1000 for all datasets (synthetic and real-word). We performed the experiments 30 times independently and in each case, we shuffled the outlier points evenly within the inlier points. Figure 1 illustrates the comparison of results between the two algorithms. Specifically, Figure 1a shows the average accuracies of C_KDE_WR and SOD_GPU, in terms of Precision, Recall and F-Score on KddCup99 dataset. Our proposed C_KDE_WR algorithm performs better in terms of Precision but slightly lower than SOD_GPU in terms of Recall score. The results on CoverType and synthetic datasets are very similar as we can see from Figure 1b–d.

Furthermore, our results show that C_KDE_WR improves over SOD_GPU in Precision and the overall F-Score on all datasets. This can be supported by the *t*-test results we have obtained in Table 1. We run the experiments and compare those metrics between these two algorithms in multiple times, with the hypothesis that the Precision and F-Score metrics of C_KDE_WR is higher than that of SOD_GPU. Based on the result in this table, we are assured that our C_KDE_WR performs better than SOD_GPU in term of accuracy over streaming context.

**Figure 2:**
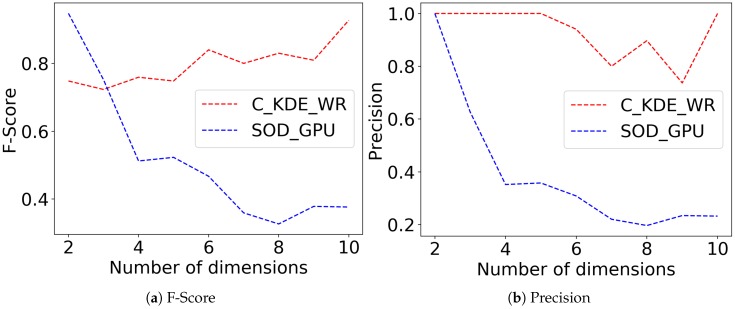
Accuracy of C_KDE_WR on synthetic data with varying dimensions.

We also compared the accuracy between C_KDE_WR and SOD_GPU algorithm as the number of data dimension increases in synthetic data. Figure 2 illustrates that C_KDE_WR demonstrates a superior performance on processing high dimensional data in both Precision and F-Score than SOD_GPU algorithm. The accuracy of our C_KDE_WR algorithm only drops slightly as the data dimension grows, while the accuracy of SOD_GPU plummets as the dimension of data increases.

### 5.5. Accuracy Evaluation for C_LOF

We test the accuracy of our C_LOF algorithm when value of *k* parameter varies. By the definition of LOF, *k* parameter is the minimum number of points in order to be considered as a cluster [12]. We set this parameter *k* from 3 to 10 and test the corresponding accuracy metrics on two synthetic datasets generated from mixture of Gaussians as per in Section 5.1.1. Note that setting *k* to 1 and 2 would not have any practical meaning and therefore we do not measure C_LOF on these two settings. Based on our experiment, the result indicates that the accuracy of C_LOF on both datasets peaks when *k* is at around 5. The precision metric stabilizes on both synthetic datasets after k=5, where it is equal to 1. However, both the recall and F-Score metrics have descended after k=5 on synthetic dataset 1, while these metrics fluctuate after k=5 on synthetic dataset 2.

**Figure 3:**
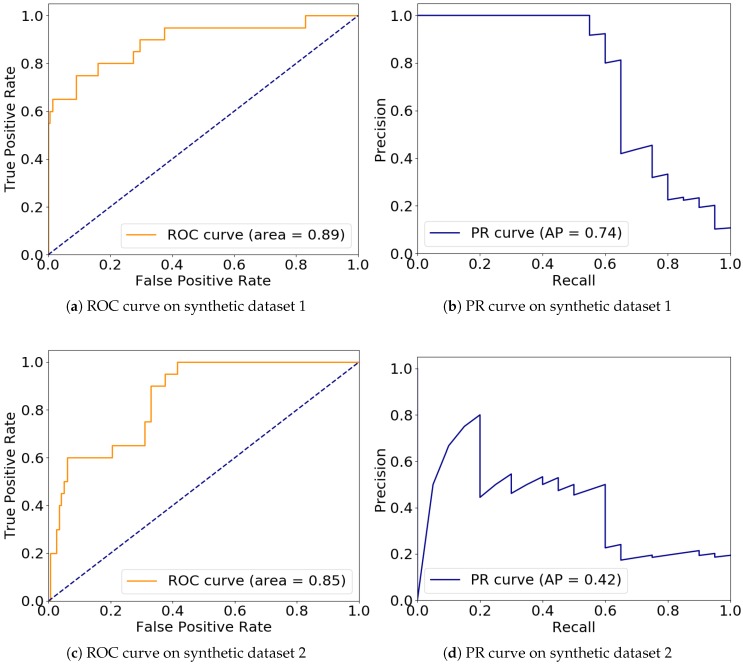
Receiver Operating Characteristic (ROC) curve and area under curve (AUC) value on synthetic dataset 1 (**a**), 2 (**c**) and PR curve and AP value on synthetic dataset 1 (**b**), 2 (**d**).

With k=5, which is the maximum parameter setting, we draw the ROC curve and PR curve on both synthetic datasets at different threshold settings in order to prove its efficiency. Figure 3 illustrates results of these metrics with also their AUC ROC values and AP (Average Precision) values shown in the figure legends.

According to the survey on outlier detection algorithms given in Reference [13], the result of our C_LOF seems descent and promising. In some cases, it is even better than some of those methods introduced in Reference [13]. Notice that the experimental results presented in Reference [13] are all conducted in a static and non-streaming environment, especially without concept drift, while result of C_LOF is run in a streaming context with the presents of both concept drift and masquerading.

## 6. Conclusions and Future Works

Our experimental results confirms that both proposed algorithms can detect outliers over data streams accurately with low number of false negatives. Based on the results of our accuracy metrics and t-test on both synthetic and real-world data, we conclude that C_KDE_WR outperforms SOD_GPU [1], which is the state-of-the-art by the time of writing, in terms of precision and overall F-score while the number of false positives is also significantly reduced. This further confirms that our novel concept of drift detection module is effective. C_LOF on the other hand, can detect outliers over streaming datasets where both concept drift and masquerading occur. Comparing its ROC AUC and AP metrics with those results presented in Reference [13], C_LOF demonstrates good efficiency in general.

We are investigating further improvements and open research directions. In particular:

- Though we managed to drop the number of false positives in C_KDE_WR, its number is still high in some specific cases. We believe that this number can be further reduced.
- The time complexity of C_LOF is still high, especially as dimension of data increases. Therefore, the result is more desirable when processing low-dimensional data. An efficient (or approximation) algorithm for clustering (based on reachability distances) is to be developed in order to decrease the overall complexity of C_LOF.
- Algorithms for detecting *Type III* outliers are barely found in the literature and therefore this area has much to be researched.

## Figures and Tables

**Table 1:** T-test: C_KDE_WR vs. SOD_GPU.

	KddCup99 Dataset		CoverType Dataset		Synthetic Dataset	
	Precision	F-Score	Precision	F-Score	Precision	F-Score
*p*-value	<$2.2\times 10^{-16}$	<$2.2\times 10^{-16}$	<$2.2\times 10^{-16}$	<$2.2\times 10^{-16}$	<$2.2\times 10^{-16}$	<$2.2\times 10^{-16}$
confidence interval	(0.106, 0.119)	(0.045, 0.055)	(0.064, 0.680)	(0.014, 0.018)	(0.264, 0.291)	(0.018, 0.056)
variance	$9.964\times 10^{-6}$	$5.669\times 10^{-5}$	$1.059\times 10^{-6}$	$2.853\times 10^{-4}$	$6.982\times 10^{-5}$	$9.941\times 10^{-5}$
